# Analysis of Biomaterials as Green Coagulants to Control Suspended Solids for Surface Water Treatment

**DOI:** 10.3390/ijerph17051777

**Published:** 2020-03-09

**Authors:** Rosa Devesa-Rey, J.D. González-Aller, Santiago Urréjola

**Affiliations:** Defense University Center, Naval Academy, University of Vigo, Plaza de España 2, 36920 Marín-Pontevedra, Spainurrejola@cud.uvigo.es (S.U.)

**Keywords:** kinetic studies, coagulation-flocculation, diatomaceous earth, lactic acid, calcium lactate

## Abstract

This study explores the use of natural, ecological coagulant-flocculants to reduce suspended particles in water. Three compounds were tested, namely: diatomaceous earth, calcium lactate and lactic acid. For this purpose, experiments in jar tests were carried out and the best compound was submitted to an optimization in order to evaluate the most significant parameters affecting its use as coagulant-flocculant. First results evidenced that lactic acid remove 71% of the suspended particles during the first five minutes, and up to 83% during the first 15 min. To optimize its use, the range of suspended particles concentration, lactic acid dose and salinity gradient was tested by means of an incomplete 3^3^ factorial design. This technique allows reducing the number of experiments to be carried out through a response surface methodology, which enables to infer the values of the dependent variables in not studied situations, by means of predictive equations. As a result of the experiments carried out, optimal conditions to remove suspended particles were set at a lactic acid concentration of 1.75 g·L^−1^. As lactic acid may be obtained biotechnologically from organic wastes, this use supposes a promising area by keeping products and materials in use and contributing to a circular economy.

## 1. Introduction

Water treatment from industrial effluents, runoff, rainfall, etc., requires a number of procedures for the removal of unwanted, potentially toxic pathogenic components. Removal of suspended particles is one of the initial stages in the water treatment, and it is fundamental for an effective purification process. Among the negative effects related to the existence of suspended particles in water are: their high photostability degree which is directly related to their persistence; they serve as a nutrient for the microorganism growth and therefore affect disinfection processes; they are responsible for the lack of water transparency which will promote eutrophication; and they also may act as carriers of contamination, as they can contain significant amount of metals adsorbed on their surface [1,2].

Removal of suspended particles is typically carried out with synthetic flocculants, which reduce or neutralize the electrostatic charges of these particles in water, favoring their aggregation and sedimentation [3]. The most employed are inorganic compounds derived from aluminium and iron salts which carry out a sweep-floc mechanism [4] which promotes the formation of positively charged Al(OH)_3_ and Fe(OH)_3_ aggregates which reacts with colloidal suspended particles, typically negatively charged, leading to their precipitation. Their use has shown high efficiency to reduce turbidity, color and organic matter. However, significant Al and Fe concentrations may remain in the treated water and achieve some unwanted toxicity. So, although the World Health Organization (WHO) recognizes their value as coagulant in water treatment, they recommend a number of approaches for minimizing their concentrations in water in order to avoid their possible neurotoxicity [5]. 

One of the alternatives for the reduction of traditional flocculants is to partially replace them with new flocculants, such as polymers or minerals [6,7]. These materials have the advantage of being more environmentally friendly, biodegradable and non-toxic. Thus, development of new materials that can be used as flocculants is an area of great interest.

This work proposes the use of three new compounds for the coagulation-flocculation process. Laboratory experiments were carried out with three different materials, namely: 

Diatomaceous earth, also referred to as diatomite, which is an accumulation of diatom (*Bacillariophyceae*) frustules, made of amorphous, porous silica, which can harvest molecules from a solution [8]. It is widely employed in the food industry to clarify reactors, and has also been employed as an adsorbent of metals in aqueous solutions and water treatment. Marthi and Smith [9] employed diatomite for the recovery of lithium from Great Salt Lake, showing a 95% of extraction with a high selectivity, although diatomite needed to be functionalized with MnO_2_ by means of a hydrothermal treatment and a solid-state calcination. Lyngsie et al. [10] employed diatomite coated with amorphous iron oxide to recover phosphate from drainage water, showing a quantitative sorption at a solution:solid ratio of 1:100. 

Calcium lactate, is a totally harmless coagulant, commonly used as a food additive (as baking powder and also to retain the firmness of table olives and other pickled vegetables), and is also added to sugar-free foods to prevent tooth decay, or as a medicine for its anti-acid behavior and to treat calcium deficiency [11]. Calcium lactate has been tested by other authors to evaluate the capacity to remove colored compounds or lignin, among others. Azreen et al. [12] tested calcium lactate for color removal from wastewater of palm oil mill effluent and the highest percentage removal observed was 80.93% at the dosage of 0.3 g/L after 20 min of treatment. Instead, the highest lignin removal achieved by Zahrim et al. [13] from aqueous solutions ranged from 50% to 68%. 

Lactic acid, named as 2-hydroxypropanoic acid following the IUPAC systematic name, was first isolated in 1780 by a Swedish chemist, Scheele, and is a carboxylic acid with a chemical formula of C_3_H_6_O_3_. In solution, it can lose a proton from the acidic group, producing the lactate ion CH_3_CH(OH)COO^−^. The main advantage of lactic acid as coagulant relies in the fact that it can be obtained biotechnologically from agricultural residues by fermentation of these substrates with lactic acid bacteria [14]. It is a compound also used in the food industry because of its biocide character and which could, therefore, expand its application spectrum. Devesa-Rey et al. [15] evaluated different materials and their efficiency was compared with AlCl_3_, one of the most common coagulants employed in the water treatment. Among the materials employed, there were tested lactic acid and calcium lactate, together with sodium lactate, citric acid, SDS, activated carbon and calcium carbonate at a selected concentration, fixed at 1 g·L^−1^ and evaluating their efficiency from 1.5 h to 5 hours. Conclusions of this study allowed determining that both lactic acid and calcium lactate gave almost the same reduction percentage of suspended solids than AlCl_3_, which was considered as a promising result in order to use these new materials in the water treatment. In the present study an optimization of their use was evaluated and it was tested the influence of their concentration and also the influence of salinity and the dose of suspended sediments. The present study evaluates, not selected wavelengths, but Nephelometric Turbidity Units (NTU) and it is aimed at reducing the time of treatments, so maximum experimentation time of 30 min will be fixed for every experiment, with intermediate evaluations at 5 and 15 min. 

Thus, in this study the overall objective is to reduce turbidity in water by means of non-traditional materials, mainly characterized by their less toxicity. For this purpose, the following individual objectives were proposed:

- To evaluate the use of different materials in order to reduce the suspended particles in water, namely: diatomaceous earth, calcium lactate and lactic acid.
- To propose the materials that best reduce the turbidity in water.
- To optimize the process of coagulation-flocculation by means of an incomplete factorial design.

## 2. Materials and Methods 

### 2.1. Materials 

**Figure 1:**
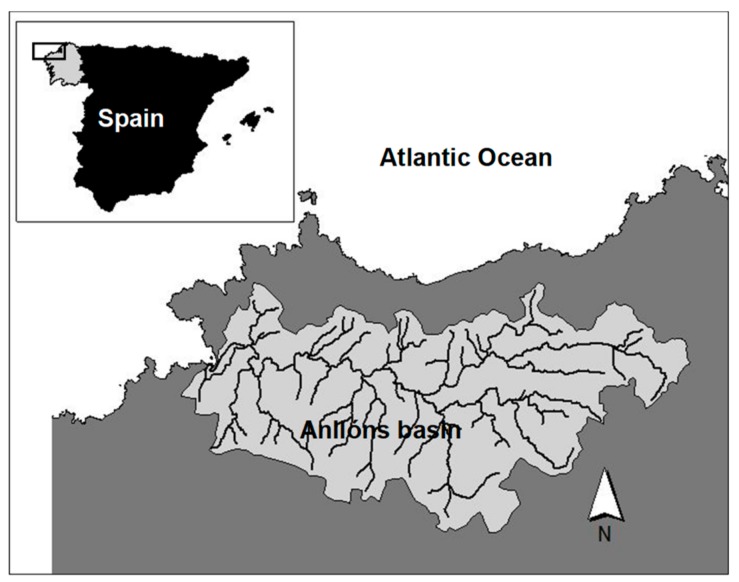
The map shows the sediment sampling area, located in the NW of Spain.

Water samples were artificially contaminated in laboratory, with sediments collected from the Anllóns River (NW Spain) (Figure 1). The collection area is a rural catchment with a history of agricultural, forestry and cattle raising activities, with a main population upstream of over 25,000 and a seafood caning industry. The river runs over schists in the upper area, turning into a smooth profile in the middle area of the river, characterized by basic rocks (gabbros and amphibolites). The lower stretch of the river runs over granite of two micas, followed by biotitic gneiss at the mouth. The land use of the area is a mixed forest of *Eucaliptus globulus*, *Eucaliptus alba* and *Pinus pinaster* (60% of the total cover), cultivated lands (18%), pastures (12%), bushes (9%) and urban uses (1%).

The sediment samples were collected with a small plastic shovel and taken to the laboratory in a hermetic plastic container, where they were freeze-dried. Only the fractions under 2 mm were considered for the experiments. Physicochemical and biological characterization of 14 sampling sites were carried out along the watercourse [16,17]. Sample selected for this study showed a sandy texture and a significant organic contamination. The main properties of the sediments employed in this study are summarized in Table 1. 

Coagulation-flocculation experiments were carried out with three different materials, namely: diatomaceous earth, lactic acid and calcium lactate. In this study, diatomaceous earth (Celatom FW-60) was first washed and then activated by means of an acid treatment with sulphuric acid (1:1 ratio) at 500 °C during 2 h, in order to remove occlusions and enhance adsorptive behavior of diatomite. 

### 2.2. Coagulation-Flocculation Experiments

**Figure 2:**
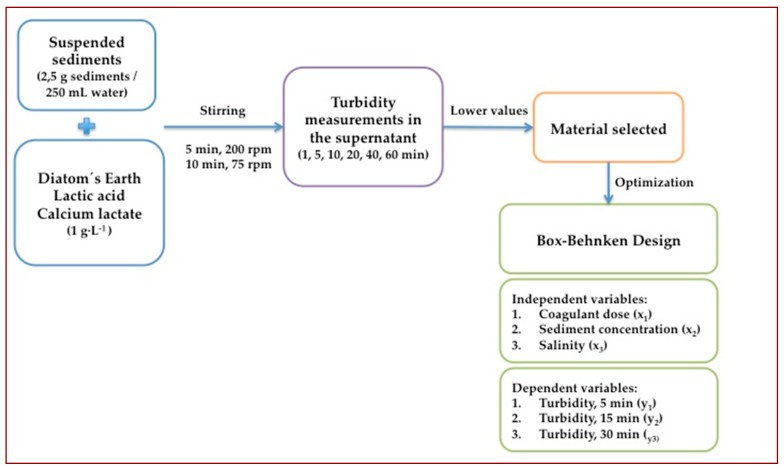
Flowchart of the process carried out.

In order to evaluate the efficiency of the coagulants tested, batch experiments were run in triplicate by placing 2.5 g of suspended solids in 250 ml of water and 1 g·L^−1^ of each coagulant. Experiments were carried out in jar tests, and the process applied to water samples was the following: stirring at 200 rpm during 5 min, followed by 75 rpm during 10 min and, after stopping agitation, surface samples were taken after 1, 5, 10, 20, 40 and 60 min and turbidity was measured (Eutech Instruments Turbidimeter TN-100, Fischer Scientific, Göteborg, Sweden). Statistical differences between results will be evaluated by means of the Student´s t-test at a 0.05 level. The material showing the highest turbidity reduction will be optimized and varying concentrations will be assayed. A 1:100 solution:solid ratio will be tested, alongside two more concentrated ratios, testing 1:50 and 1:33. Figure 2 illustrates the process carried out in this project. 

### 2.3. Box–Behnken Experimental Design

Based on the results of the previous procedures, the best coagulant-flocculant will be selected and an optimization of its use will be carried out. The variables chosen for the optimization of the process were: coagulant-flocculant concentration, suspended particles concentration and salinity effect, as this is a factor that may be relevant in the treatment of water from seawater. 

The combination of variables to achieve the highest suspended solids removal was carried out by means of a response surface methodology. This technique consists of a group of mathematical and statistical techniques based on fitting empirical models to the experimental data obtained in relation to experimental design [18]. Box–Behnken designs are a class of rotatable or nearly rotatable second-order designs based on three-level, incomplete factorial designs [19]. The number of experiments (N) required for full Box–Behnken design are given by the formula:

(1) $$N=2k\left(k-1\right)+C_{0}$$

where k is the number of factors and C_0_ is the number of central points [20]. The simplest equation describing a linear function is described by Equation (2):

(2) $$y=\beta_{0}\sum_{i=1}^{k}\beta_{i}X_{i}+\varepsilon$$

where *β_0_* is the constant factor; *β_i_* represents the coefficients of the linear parameters; *k* is the number of variables; *x_i_* represents the variables, and ε is the residual factor associated with the experiments. When the experimental data do not fit a linear equation, then it is desirable to include levels in the input variables. In this case, a polynomial response surface must be generated. Box–Behnken experimental designs were constructed for situations in which it is desirable to fit a second-order model (Equation (3)).

(3) $$y=\beta_{0}\sum_{i=1}^{k}\beta_{i}X_{i}+\sum_{i=1}^{k}\sum_{j\geq i}^{k}\beta_{ij}X_{i}X_{j}+\varepsilon$$

where *β_ij_* represents the coefficients of the interaction parameters. These designs include a central point used to determine the curvature, and critical or optimal conditions are deduced from the above second-order function by including quadratic terms (Equation (4)).

(4) $$y=\beta_{0}\sum_{i=1}^{k}\beta_{i}X_{i}+\sum_{i=1}^{k}\beta_{ii}X_{i}^{2}+\sum_{i=1}^{k}\sum_{j\geq i}^{k}\beta_{ij}X_{i}X_{j}+\varepsilon$$

where *β_ii_* represents the coefficients of the quadratic parameters. Thus, the experimental data enable the development of empirical models that describe the interrelationship between operational and experimental variables by equations including linear, interaction and quadratic terms. 

Thus, the quadratic function obtained for all three variables is described in Equation (5).

(5) $$y=\beta_{0}+\beta_{1}X_{1}+\beta_{2}X_{2}+\beta_{3}X_{3}+\beta_{12}X_{1}X_{2}+\beta_{23}X_{2}X_{3}+\beta_{23}X_{2}X_{3}+\beta_{11}X_{1}^{2}+\beta_{22}X_{2}^{2}+\beta_{33}X_{3}^{2}\;\;$$

where *y* is the dependent variable, *β* denotes the regression coefficients (calculated from experimental data by multiple regressions using the least-squares method) and *x* denotes the independent variables. The experimental data were analyzed by the Response Surface method with Statistica 7.0 software (Tibco, Palo Alto, USA).

## 3. Results and Discussion

### 3.1. Evaluation of Three Coagulant-Floculants 

**Figure 3:**
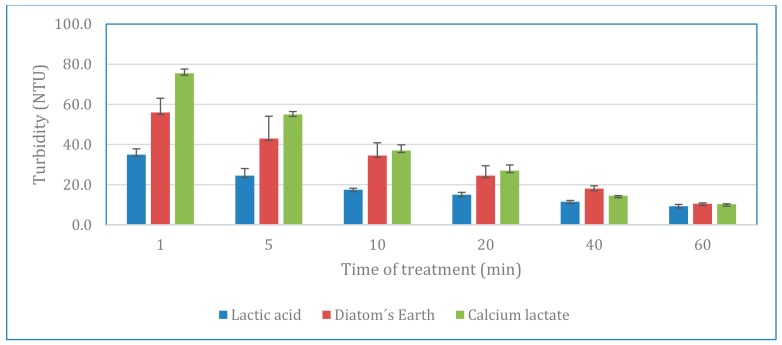
Turbidity results for the three coagulant-flocculants tested. Bars indicate the variation coefficient of for each experiment.

From the data obtained it was observed the higher turbidity reduction for lactic acid, which showed a value of 35.0 NTU in the first minute of treatment. Calcium lactate and diatomaceous earth reached a similar value after 10 min. So, turbidity of wastewater treated with lactic acid was a 37.5% and 54% lower than the showed by diatomaceous earth and calcium lactate, respectively, after the first minute of treatment. Similar behavior was observed after 5 to 20 min of treatment. After 40 min, differences between the three materials were less marked, with values ranging from 11.5 to 14.5 NTU. Finally, after 60 min of treatment there are no significant differences between lactic acid, diatomaceous earth and calcium lactate. Results of turbidity reduction are shown in Figure 3. 

The use of lactic acid in the water treatment is an area of great interest from an environmental point of view. It can be obtained from residues, as the organic fraction of municipal solid wastes [21], which show a great bioconversion potential and thus the negative effects of its disposal can be then avoided. These authors estimated that 0.23 g of lactic acid could be produced from one gram of dry organic fraction of municipal solid waste. Morrone [22] analyzed the possible pathways to recover residual biomass resources, including tomato pomace agro-food residues as a source of lactic acid. Additionally, this compound may be biotechnologically obtained from wastes with high content of hemicelluloses. González-Leos [23] found that lactic acid could be obtained from sugarcane bagasse hydrolisates, fermented with *Lactobacillus pentosus*, obtaining up to 55.437 g·L^−1^ of lactic acid. Moreover, Soltanian et al. [24] presented a survey to investigate a lignocellulosic-based sugarcane biorefinery with cogeneration of lactic acid and power. The 20.49% of the total cost of the biorefinery was due to the lactic acid synthesis unit. So, waste recycling for lactic acid cogeneration in a biorefinery and its use in water treatment contributes to sustainability, and it enhances the concept of a regenerative economy by keeping products and materials in use, reducing wastes and pollution and contributing to circular economy. 

### 3.2. Model Statistical Testing

Based on these results, the best coagulant-flocculant was selected by means of an incomplete 3^3^ factorial design. The variables chosen for the optimization were: coagulant-flocculant concentration, suspended particles concentration and salinity effect, as a factor that may be relevant in the treatment of seawater. For this purpose, 15 samples were prepared, in which the quantities of sediments, salinity and dose of coagulant were varied. The experimental design includes three central points (experiments 13−15) fixed at the average concentrations of each variable, to evaluate the quality of the experimental process. As in the previous process, the samples were subjected to the following stirring scheme: 200 rev/min for 5 min and 75 rev/min for 10 min. Turbidity measurements in the supernatants were made after 5, 15 and 30 min of sedimentation.

The incomplete 3^3^ factorial design was used to elucidate the optimal conditions of the three variables assayed in reducing the turbidity caused by suspended sediments in water samples. The experimental conditions assayed for the independent variables (expressed in terms of coded variables) are shown in Table 2. The standardized (coded) dimensionless independent variables used, with variation limits (-1, 1), were defined as x_1_ (concentration of the selected coagulant), x_2_ (sediment to water ratio) and x_3_ (salinity).

In order to simulate turbidity in water, riverbed sediments were employed to prepare suspensions and maximum turbidity values, expressed as Nephelometric Turbidity Units (NTU) were above 100. Dependent variables analyzed in this study were turbidity reduction after 5, 15 and 30 min, which corresponded to variables y_1_, y_2_ and y_3_, respectively (Table 3). 

In order to evaluate the influence of each independent variable in the turbidity reduction, 15 experiments were carried out. Experiments 13−15 were replicates of the central point of the design, employed as statistical control to evaluate the experimental error. Operational conditions assayed, as well as results obtained are shown in Table 4. Results obtained showed different degrees of efficiency, with turbidity values ranging from 31 to 98 NTU, after 5 min of experiment; from 29.2 to 88.1 after 15 min; and from 20.2 to 60.1 NTU after 30 min. So, whereas some experiments showed negligible effects in turbidity reduction, others showed a reduction above 70% during the first 5 min. Analysis of replicates of the central point (experiments 13−15) showed a low variability; with variation coefficients lower than 10% in all cases. 

**Figure 4:**
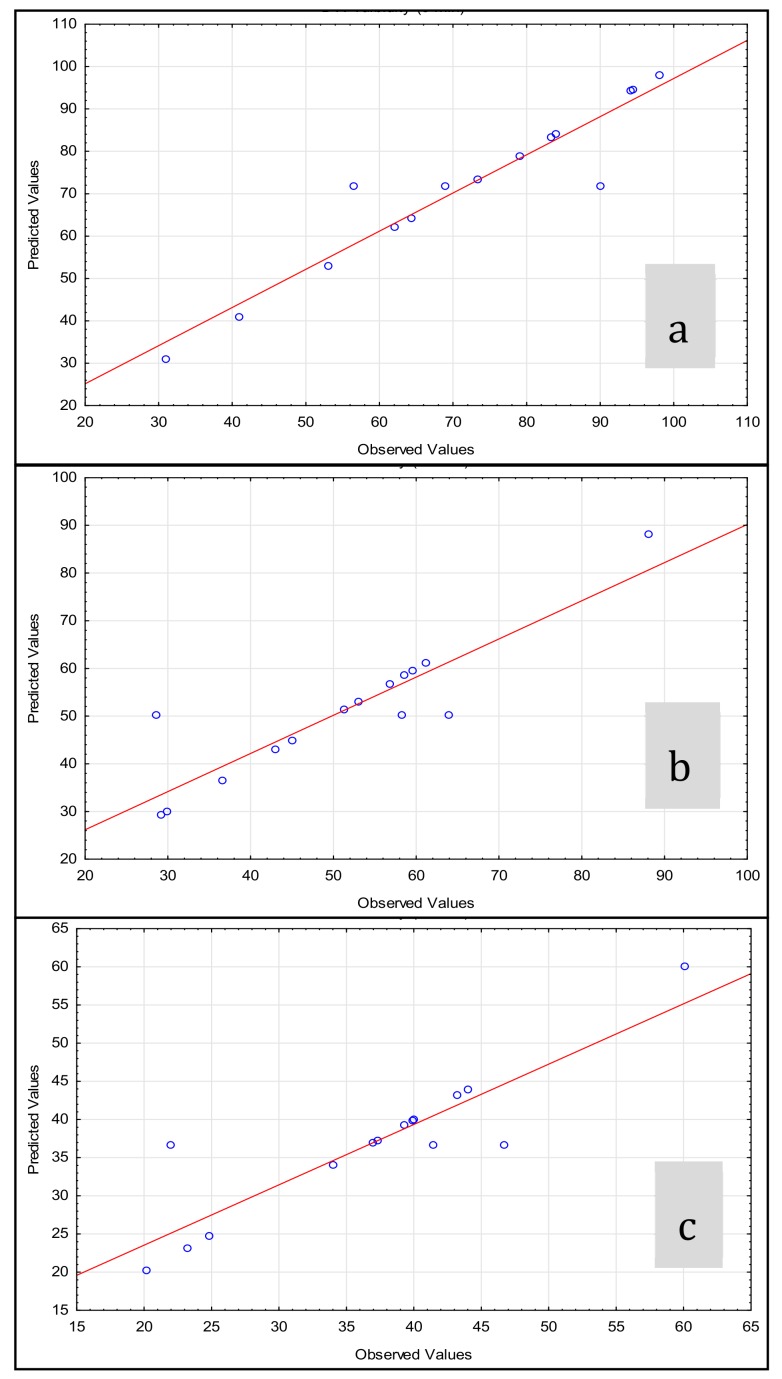
Observed of predicted values for the experimental design. (**a**) Results for dependent variable, y1; (**b**) results for dependent variable, y_2_; (**c**) results for dependent variable, y_3_.

The fitting of the model can be evaluated comparing the predicted data, which is plotted as a line fitted in linear regression. Observed data are plotted as blue dots in Figure 4 and comparison among predicted vs. observed data against the 1:1 line suggest an appropriate fitting of the model. Observed data fits within the 90% prediction interval, so no outliers were found within experimental data obtained. 

**Figure 5:**
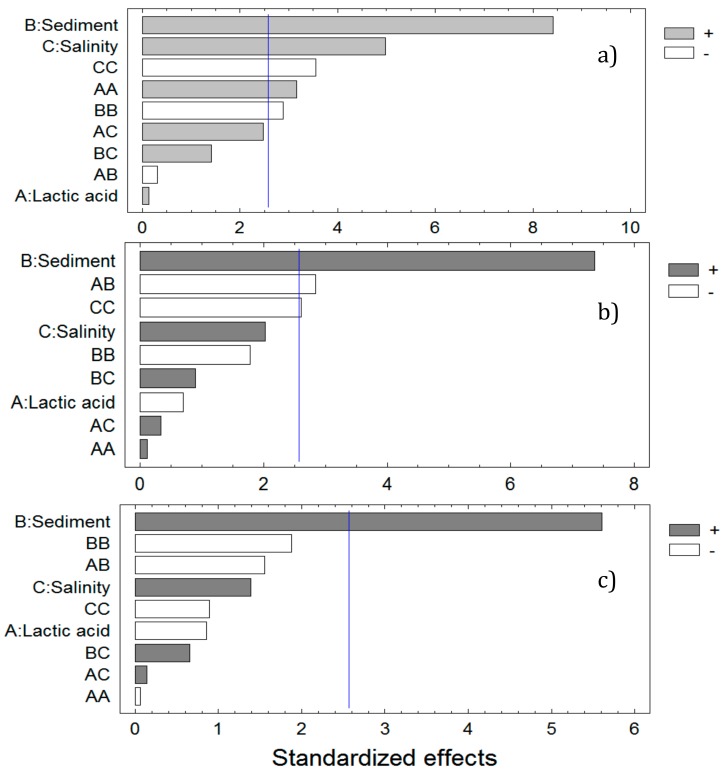
Standardized Pareto graphs showing single and interaction effects for turbidity reduction after (**a**) 5 min, (**b**) 15 min and (**c**) 30 min.

The following step was to evaluate which variables are the ones that most influence the turbidity reduction process. For this purpose, Pareto charts were plotted (Figure 5). Their left vertical axes represent the independent variables assayed, as single, interaction or quadratic factors in decreasing order. The purpose of the Pareto chart is to highlight those factors significantly influencing the turbidity reduction. Parameters exceeding the acceptance limit represented by blue line are considered relevant in the process. 

Figure 5 shows standardized Pareto charts for variables influencing turbidity reduction after 5, 15 and 30 min. During the first 5 min of treatment, variables more influencing turbidity reduction are sediment concentration and salinity as well as interaction effects between salinity, sediment concentration and lactic acid. After 15 min only sediment concentration appears as the most significant factor affecting turbidity reduction, together with interaction effect of lactic acid and sediment (AB factor). At minute 30 only sediment concentration showed significance in water treatment. 

**Figure 6:**
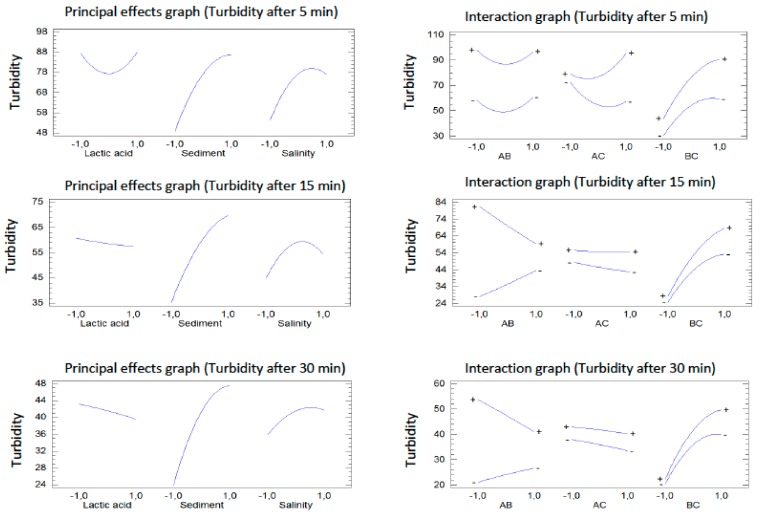
Principal and interaction effects for the three independent variables studied. Interaction graph denotes interaction among variables (A: lactic acid; B: sediment; C: sediment).

According to the association observed between the three independent variables analyzed in this study, a closer relationship between them was analyzed. So, principal and interaction effects were evaluated (Figure 6). Principal effects graphs show an increasing turbidity with suspended sediments concentration, whereas the highest lactic acid concentration shows a benefit in the water treatment, reducing turbidity. Salinity shows a negative effect, with the lowest turbidity values shown at the minimum salinity concentration. This behavior does not obey the salting-out effect, which explains the precipitation of solutes under high ionic strength. However, other authors have also observed the highest precipitation at the lowest salinity. Accordingly, Chandra et al. [25] found that saline water needed higher times to reach equilibrium concentration on the evolution of suspended sediment concentration. In their study about the effect of salinity on suspended cohesive sediments, Gudrum et al. [26] found that silica sediments deposit faster with zero salinity than in saline water. Additionally, Shadrin [27] also found a significant increase in total suspended solids and dissolved organic matter with increasing salinity. 

After the analyses of Pareto and principal effect graphs, predictive equations were formulated, taking into account only the significant coefficients (at *p* < 0.05). Predictive equations include single, interaction and quadratic effects. Equations (6)–(8) may serve as proxies for the turbidity reduction due to suspended sediments in water, as a function of the initial sediment concentration, salinity and lactic acid concentration. The equations are shown below:

(6) $$Turbidity,\;5\;min=77.3+18.9\left[Sed\right]+11.2\left[Sal\right]+10.5{\left[Lactic\right]}^{2}-9.6\left[Sed\right]-11.8\left[sal\right]$$

(7) $$Turbidity,\;15\;min=58.7+17.2\left[Sed\right]-9.4\left[Lactic\right]\left[Sed\right]-8.9{\left[Sal\right]}^{2}$$

(8) $$Turbidity,\;30\;min=41.5+1.7\left[Sed\right]$$

**Figure 7:**
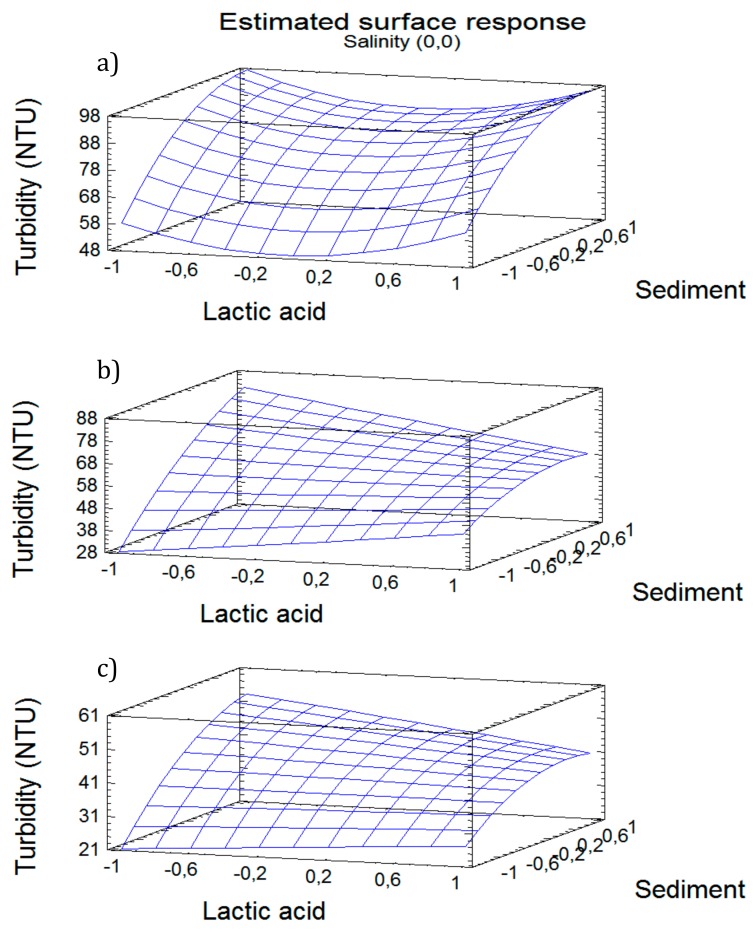
Variation in turbidity with lactic acid and sediment concentration, at different times: (**a**) 5 min; (**b**) 15 min; (**c**) 30 min.

Equations (6)–(8) can be employed to predict the optimum combination of variables leading to the lowest turbidity. Variation in turbidity with lactic acid and sediment concentrations, after 5, 15 and 30 min of treatment are shown in Figure 7. Results obtained evidenced the need to use a quadratic model instead of a linear model, since the relationship among variables does not vary linearly. The response surface obtained showed a curvature that suggests the data must be modeled by including quadratic terms, which will allow for the mapping of a region of response surface, in order to interpret it and to select the operating conditions that must meet the specifications required.

According to the optimization studies carried out, the model predicts that 29 NTU can be reached after 5 min with an optimal combination of lactic acid (+0.313 coded value), sediment (-1.0 coded value) and salinity (-1, coded value). After 15 min, turbidity can reach the minimum value of 18 NTU, with a combination of lactic acid:sediment:salinity of (+0.99:-1.0:-0.97), expressed as coded values. After 30 min of experimentation, turbidity reduction is negligible, as the model predicts that 17 NTU can be reached, with a combination of (+1.0:-0.99:-1.0), expressed as coded variables. 

There is a growing concern about the employment of ecological and biodegradable flocculants for wastewater treatment, which represent many advantages for being natural, renewable, non-toxic and biodegradable [28]. Clarification of river water to produce potable water requires a treatment to remove suspended particles, as suspended particles act as carriers of metal contamination [16,29,30,31] due to their high surface area. Suspended particles regulation in water is considered necessary in order to flood control security, to keep the socio-economic benefit of reservoirs, hydropower production and the water supply [32]. The use of lactic acid for the coagulation-flocculation of particles in water is a promising area since this compound is becoming the target of waste bio-valorization and this may convert it into highly available at a moderate cost. Xu et al. [33] produced lactic acid from food waste and waste activated sludge by batch fermentation with a productivity rate of 0.53 g·L^−1^·h^−1^. Solid vinasses from liquor industries may contain up to 106 g of lactic acid per kg [34] and so therefore vinasses residues are another important source for lactic acid production [35]. The organic fraction of municipal solid waste may turn into an important source and López-Gómez et al. [36] point at its importance in this bioeconomy model. The use of agricultural and agro-industrial wastes and by-product streams will be an important lactic acid source [37] and it will bring support to reduce the overall production cost, aiming to bridge the waste suppliers to the chemical production industries and it will help to incorporate waste from other sectors into the life cycle of a new compound with proven efficiency in the water treatment.

On the other hand, the use of chemometric tools has proved to be very useful for the quick determination of the most influential variables in the turbidity reduction which is also estimated with the analysis of variance (ANOVA) so only factors with a statistical significance will be selected for the model. Response surface methodologies results very useful to obtain the critical conditions of the factor (maximum or minimum) and, particularly, Box–Behnken designs (BBD) show a high degree of efficiency [38]. Other authors have validated the use of incomplete factorial designs, such as BBD, which allow estimation of first and second order terms in the model, but requires fewer experiments when compared with a Full Factorial Design (FFD), which studies the effects of all combinations of the possible levels including the main and interaction effects, while testing the model curvature. 

## 4. Conclusions

Lactic acid and calcium lactate use as coagulant-flocculants was optimized, as they may be an interesting alternative to aluminium salts in the water treatment. As treatment with 1 g·L^−1^ had already given promising results, optimization allows determining the coagulant dose, the time of treatment and the influence of salinity. Comparison between the three materials employed—diatomaceous earth, calcium lactate and lactic acid—showed the highest turbidity reduction for lactic acid. When its use was optimized by means of an incomplete 3^3^ factorial design it was observed a turbidity reduction of 71% during the first five minutes of experiment, whereas after 15 min, turbidity reduction reached the 83%. Calcium lactate and diatomaceous earth showed lower turbidity reductions in the range tested. Thus, lactic acid appears as an ecofriendly alternative in the use of coagulant-flocculants in water treatment, as it may be biotechnologically obtained from lignocellulosic residues and then employed to reduce suspended sediments in water. Additionally, using factorial designs results very usefully to reduce the number of experiments and to achieve conclusions in a shorter period of time. Finally, results obtained will allow the testing of lactic acid at a large scale. 

## Figures and Tables

**Table 1:** Characterization of sediment employed to contaminate water samples in the present study.

Parameter	Value	Parameter (mg·kg^−1^)	Value
Lithology	Gabbros	Cr	284.8
Textural classification	Sandy	Fe	7.8
Total Organic Carbon (TOC) (%)	10.60	Cu	68.3
Nitrogen (%)	0.84	Zn	287.3
Total Phosphorus (mg·kg^−1^)	2,324	Total chlorophyll	114.8
Available Phosphorus (mg·kg^−1^)	877		
C/N ratio	13		
N/P ratio	36		

**Table 2:** Independent variables analyzed in the coagulation-flocculation process for selected coagulant.

Independent Variables			
Variable	Nomenclature	Units	Range of Variation
Lactic acid concentration	[Lactic]	g·L^−1^	0.5−2.5
Sediment concentration	[Sed]	%	2.5−7.5
Salinity	[S]	g·L^−1^	0−33
Adimensional coded variables			
Variable	Nomenclature	Units	Range of variation
Dimensionless lactic acid concentration	x_1_	([Lactic]-3)/1.5	(-1, 1)
Dimensionless sediment concentration	x_2_	([Sed]-10)/5	(-1, 1)
Dimensionless salinity	x_3_	([S]-49)/24.5	(-1, 1)

**Table 3:** Dependent variables determined in the wastewater analyzed.

Dependent Variables		
Variable	Nomenclature	Units
Turbidity, 5 min	y_1_	NTU
Turbidity, 15 min	y_2_	NTU
Turbidity, 30 min	y_3_	NTU

**Table 4:** Operational conditions considered in the study (expressed as coded independent variables) and experimental results completed for the dependent variables assayed, x_1_ (lactic acid concentration; g L^−1^), x_2_ (sediment concentration; g L^−1^) and x_3_ (salinity).

Independent				Dependent		
Exp.	x_1_	x_2_	x_3_	y_1_	y_2_	y_3_
1	0	-1	-1	31.0	29.2	23.2
2	0	1	-1	62.0	51.3	37.3
3	0	-1	1	41.0	29.9	24.8
4	0	1	1	90.0	63.9	46.7
5	-1	-1	0	56.5	28.6	22.0
6	-1	1	0	94.2	88.1	60.1
7	1	-1	0	64.3	36.6	20.2
8	1	1	0	98.0	58.5	39.9
9	-1	0	-1	73.3	43.0	34.0
10	-1	0	1	83.3	53.0	39.3
11	1	0	-1	53.0	45.0	37.0
12	1	0	1	94.5	59.5	44.0
13	0	0	0	84.0	61.1	40.0
14	0	0	0	79.0	56.8	43.2
15	0	0	0	69.0	58.2	41.4
